# Lollipop containing *Glycyrrhiza uralensis* extract reduces *Streptococcus mutans* colonization and maintains oral microbial diversity in Chinese preschool children

**DOI:** 10.1371/journal.pone.0221756

**Published:** 2019-08-23

**Authors:** Yandi Chen, Melissa Agnello, Márcia Dinis, Kenneth C. Chien, Jing Wang, Wei Hu, Wenyuan Shi, Xuesong He, Jing Zou

**Affiliations:** 1 State Key Laboratory of Oral Diseases, National Clinical Research Center for Oral Diseases, West China Hospital of Stomatology, Sichuan University, Chengdu, China; 2 School of Dentistry, University of California, Los Angeles, Los Angeles, California, United States of America; 3 School of Dentistry, University of California, San Francisco, San Francisco, California, United States of America; 4 College of Pharmaceutical Science, Shandong University of Traditional Chinese Medicine, Jinan, China; 5 State Key Lab of Microbial Technology, Microbial Technology Institute, Shandong University, Qingdao, China; 6 The Forsyth Institute, Cambridge, Massachusetts, United States of America; Fred Hutchinson Cancer Research Center, UNITED STATES

## Abstract

The anticariogenic activity of the extract of *Glycyrrhiza uralensis* (licorice) has been well documented. We recently developed an herbal lollipop containing licorice extracts with Glycyrrhizol A, the compound displaying strong antimicrobial activity against *Streptococcus mutans*. Preliminary testing showed that the herbal lollipop reduced salivary *S*. *mutans* counts *in vivo*. In this study, we aimed to further test the efficacy of this herbal lollipop for reducing salivary *S*. *mutans* levels, and investigate its impact on salivary microbiome. Using a well-established *in vitro* oral microbiome model, we showed that licorice extract displays targeted killing against *S*. *mutans* without affecting the biodiversity of the community. *In vivo* study corroborated *in vitro* findings, showing for high caries-risk children aged 3–6 with salivary *S*. *mutans* levels >5x10^5^ cells/ml, daily use of 2 licorice-containing lollipops for 3 weeks significantly reduced salivary *S*. *mutans* levels compared to the control group. Salivary microbiome analysis showed either no change or even increase in phylogenetic diversity of the oral community following herbal lollipop usage. Although further study with longer term observation is needed, these results suggest that use of licorice extract-containing lollipops can be as a simple and effective way to reduce the risk of dental caries in children.

## Introduction

Dental caries is a chronic, infectious and highly prevalent disease throughout the world. Young people, especially children, are primarily affected. Early childhood caries (ECC), defined as the presence of one or more decayed (noncavitated or cavitated lesions), missing (due to caries), or filled tooth surfaces in any primary tooth in a child 71 months of age or younger, causes worldwide concern due to its negative impact on children’s overall health and well-being[1, 2]. From 1987 to 2013, the pooled national prevalence and care index (ft/dmft%) for ECC in mainland China were 65.5% and 3.6%, respectively[3]. Inappropriate feeding practices (especially misuse of baby bottles), poor knowledge of oral hygiene and dental care among parents, and low dentist-to-population ratio (1:10000), are common reasons why Chinese children suffer greatly from dental caries. The current situation is far from the target set by the WHO in 2000 for 50% of children at age 6 to be caries-free[3, 4].

Known as a multifactorial, diet-dependent disease with genetic and behavioral susceptibility components, the four main etiological factors of dental caries include: (1) cariogenic bacteria, (2) fermentable carbohydrates, (3) a susceptible tooth and host and (4) time[5]. The fermentation of dietary carbohydrates, especially sucrose, by cariogenic bacteria produces organic acids, and in a fragile tooth structure, will directly result in demineralization and tooth decay[6]. *Streptococcus mutans* is considered the principle causative organism in the initiation and progression of dental caries due to its acidogenicity, aciduricity, insoluble glucan production and other virulence factors, which have been extensively studied[6–8]. Salivary *S*. *mutans* levels have been previously used as a risk indicator of dental caries; as subjects with higher levels of *S*. *mutans* have been shown to develop more caries than those with lower levels[9, 10].

Although preventative measures against caries, such as the use of fluoride, pit and fissure sealants, antibiotic agents and probiotics, have greatly contributed to a reduction in caries prevalence[11–13], simpler preventive techniques would encourage more widespread use and enhance accessibility. As a high sugar diet is a significant factor contributing to caries risk, an ideal solution would reduce sugar intake from food sources as well as inhibit bacterial growth.

The root of plant *Glycyrrhiza uralensis*, also known as licorice is an important traditional Chinese herbal medicine and is widely used as a sweetening agent in mainland China and many other countries. The potential beneficial effects of licorice in oro-dental diseases, including caries, have already been reported[14]. The compound Glycyrrhizol A, an ethanolic extract from licorice, has been shown to exhibit strong antibacterial activity against *S*. *mutans*[15]. A lollipop containing Glycyrrhizol A was developed by our group as a potential novel caries preventative method, and has been tested both *in vitro* and in a cohort of children to assess its efficacy in reducing salivary levels of *S*. *mutans* [16, 17]. While these herbal lollipops were effective in reducing *S*. *mutans* counts both *in vitro* and *in vivo*, its potential impact on the overall microbial structure still remained to be investigated. The aim of the current study was to further test the efficacy of licorice extract *in vitro* using a well-established oral multispecies microbial community model, and *in vivo* with a cohort of Chinese preschool children at high caries risk. We investigated the efficacy of a licorice extract-containing herbal lollipop in reducing salivary *S*. *mutans* levels, as well as its impact on the salivary microbiome.

## Materials and methods

### Plant material and production of herbal lollipop with licorice extracts

The detailed information on the collection and identification of the roots of Glycyrrhiza uralensis (licorice), as well as the deposition of a voucher specimen (deposited in the school of dentistry, University of California, Los Angeles with reference number GS-Jiangying-002) has been reported previously[15]. The procedure for the production of licorice extracts, the chemical analysis of glycyrrhizol A within licorice extract, as well as the production of sugar-free herbal lollipop containing licorice extracts (with one lollipop (10g) containing 10mg of licorice extract), has been extensively described[16].

### MIC testing

To determine the minimum inhibitory concentration (MIC) of licorice extract against *S*. *mutans*, susceptibility testing was performed using the broth microdilution method according to guidelines by CLSI [18]. Licorice extract was prepared as described [16]and tested in 2-fold dilutions in concentrations ranging from 12.8 mg/ml to 0.0125 mg/ml against *S*. *mutans* UA140 reference strain.

### Growth curve and kill assays

The licorice extract effect on *S*. *mutans* UA140 growth was tested. *S*. *mutans* overnight cultures in brain heart infusion (BHI, BD Biosciences) were diluted to OD600 of 0.05–0.01. Solutions of licorice extract in PBS at the concentrations 25 μg/ml, 50 μg/ml, or an equal volume of PBS (negative control), was added to the cultures at the initial time point (T0). Cultures were grown at 37°C in microaerophilic conditions (2% oxygen). At each time point, hourly for 12 h, an aliquot of culture (100 μL) was collected and optical density was measured at 600nm using a microplate absorbance reader (iMark™ Microplate Absorbance Reader, BioRad). Assay was performed in duplicate.

To perform the kill assay, *S*. *mutans* UA140 was grown in 45 mL of BHI to OD_600_ of 0.8. The culture was spun down at 5000 rpm at 4°C for 10 minutes, supernatant discharged and pellet washed twice with PBS, before being resuspended in 45 mL of PBS. An aliquot of 1mL was removed for initial time point (T0), CFU counts. The culture was then split into 3 separate cultures of 15 mL each, spun down and resuspended in 25 μg/ml licorice extract dissolved in PBS, 50 μg/ml licorice extract dissolved in PBS, or an equal volume of PBS alone. At each time point (5 min, 15 min, 30 min, 1 h, 3 h and 6 h) after bacteria cells were pelleted and resuspended, an aliquot of 1mL was collected, immediately spun down, washed 3 times with PBS, and serially diluted and drop-plated for CFU count. Assay was performed in duplicate.

### “*S*. *mutans*- enhanced” multispecies culture and kill assay

A mixed microbial community derived from a previous study [19]was cultured overnight under microaerophilic conditions (2% oxygen) in SHI media, a specialized medium previously shown to maintain over 80% of oral species diversity[19]. *S*. *mutans* UA140 was grown in BHI to exponential phase at OD_600_ of 0.8–1.0 and 10 mL was added to 40 mL of the community culture in SHI media supplemented with 0.5% sucrose.

The kill assay was performed on the prepared *in vitro* oral microbial community spiked with *S*. *mutans*, as described above, with the addition of licorice extract to a final concentration of 25 μg/ml. Cultures were incubated in microaerophilic condition at 37°C and samples were taken periodically during an overnight regrowth period. Briefly, at each time point two aliquots were collected, spun down, washed twice with PBS to remove the licorice extract, and re-suspended in equal volume of PBS. One aliquot was used for serial dilutions and drop plating for CFU assessment. In order to accurately assess which species survives or dies after treatment, the second aliquot was re-grown overnight in 3mL of fresh SHI media supplemented with 0.5% sucrose under microaerophilic conditions. This regrowth was done in order to limit the carryover of DNA from dead bacteria affecting downstream PCR and sequencing.

Afterwards, aliquots from regrown cultures at each time point were collected, cells were spun down and immediately frozen at -80°C for DNA extraction. Total genomic DNA from bacterial cultures, as well as saliva samples were isolated using the MasterPure™ DNA purification kit (EPICENTRE, Madison, WI, USA). DNA quality and quantity were measured by a UV spectrophotometer at 260 nm and 280 nm. (Spectronic Genesys™, Spectronic Instrument, Inc. Rochester, New York, USA), and used for qPCR and 16S sequencing analysis as described below.

### Quantitative PCR (qPCR)

The *S*. *mutans* and total bacteria levels were assessed by qPCR. Species-specific primers (gtf-F: CTACACTTTCGGGTGGCTTG, and gtf-R: GAAGCTTTTCACCATTAGAAGCTG) for *S*. *mutans* glucosyltransferases (gtfB) gene, and universal primers (Eub338: ACTCCTACGGGAGGCAGCAG; and Eub518: ATTACCGCGGCTGCTGG) for total bacteria was used to quantify *S*. *mutans* and the total bacteria, respectively.

Real-time PCR was performed on the Bio-Rad iCycler Thermal Cycler with iQ5 Multicolor Real-Time PCR Detection System (BioRad iQ 5 RTPCR QPCR). Two μL of DNA (15ng/uL) or 1:10 dilution for total bacteria detection, was used as template, 1μL of each primer (10uM) were mixed with SYBR Green Master Mix (BioRad). Distilled water was added to a final volume of 20 μL. Amplification was carried out as follows: initial denaturation for 4 min at 95°C, 39 or 40 cycles of denaturation for 30 seconds (s) at 95°C, primer annealing for 30 s at 64°C for gtfB and universal primers, and extension for 30 s at 72°C. All amplifications and detections were carried out on the BioRad iQ 5 RTPCR QPCR 96-wells reaction plate with optical caps (BioRad), and were performed in triplicate.

To generate standard curves for real-time PCR, tenfold serial dilutions of *S*. *mutans* DNA were used as template. The threshold at the different dilution points was averaged. Standard curves from different bacterial strains were generated with real-time PCR, and linearity with the universal primer was found.

### Ethical review and informed consent

The clinical study protocol was approved (approval number: WCHSIRB-D-2016-186) by the Research Ethics Committee of West China School of Stomatology, Sichuan University and all methods were performed in accordance with relevant guidelines and regulations. A written informed consent was signed by all children’s parents or guardians. Children were provided dental treatment as needed.

### Clinical study design

A total of 96 children aged between 3 and 6 were recruited in the screening visit. Children with systemic diseases, with periodontitis or other oral mucosal diseases, with allergies to licorice and other ingredients, had antibiotics in the past 3 months, had prescriptive or non-medications, or antimicrobial mouthwash that may influence the results were excluded. A total of 37 high-risk children, defined by >5x10^5^ *S*. *mutans* cells per ml of saliva determined by antibody-based method (refer to following sections) were enrolled in the study, 20 boys and 17girls, with an average age of 4 years 10 months. Subjects were divided into the treatment and control groups. Twenty-three children were included in the treatment group, which received lollipops and oral health care counseling; while the other 14 children received oral health care counseling only as control group. Children in the treatment group were asked to consume two lollipops a day (one in the morning after brushing teeth, and the other at night, at least 30 min before brushing teeth) for three weeks. No placebo was used in the control group because no parents or caregivers wanted their children to use products without potential benefit. Thus, children in the control group had parallel visits without lollipops (these children were informed that they would receive the lollipops as rewards after the program was completed). There were no restrictions on food or drinks for children in both groups in order to maintain their daily dietary habits. Lollipops used in this study were produced as previously described[16].

### Saliva sampling and oral examination

Unstimulated saliva (at least 2ml) was collected from each participant between 10 a.m—12 p.m by expectorating into 15ml sterile plastic tubes (Corning Centristar). Afterwards 1ml was used for quantifying salivary *S*. *mutans* level using antibody as described in the following section and the other 1ml was stored at -80°C for subsequent DNA extraction and sequencing. All children were fasting or had not eaten for at least 2 hours prior to the visit, and we made sure that they had morning tooth brushing. An oral examination was performed by an experienced pediatric dentist according to the criteria outlined by WHO, and dmft/dmfs as well as soft tissue status were recorded.

For the treatment group, sample collections were taken at the baseline visit (before lollipop uptake), 1 week, 2 weeks, 3 weeks after initial lollipop treatment, and in a follow-up visit 1 week after the termination of lollipop use. Similarly, control group samples were taken at the same time point as the treatment group. Salivary *S*. *mutans* levels at each time point from both groups were determined by antibody-based approach. Saliva samples taken from the baseline visit and 3 weeks following initial treatment were subject to DNA isolation as described above and used for 16s rRNA gene sequencing.

### Detection and quantification of salivary *S*. *mutans* levels

To minimize viable cell loss, samples were fixed in 1% formaldehyde after saliva collection and shipped to Shandong University, where a species-specific monoclonal antibody (MAb) against *S*. *mutans* was used in conjunction with fluorescent microscopy and flow cytometric techniques to detect and quantify the level of *S*. *mutans*[20].

### 16S rRNA gene sequencing and analysis

Extracted DNA from both *in vitro* cultures and saliva samples were sent to the UCLA Microbiome Sequencing Core for library prep and Illumina sequencing of the amplified V3 to V4 16S DNA region. QIIME (Quantitative Insights into Microbial Ecology) version 1.9.1 [21]was used for analysis of sequencing data. Sequences were clustered into operational taxonomic units (OTUs) using UCLUST [22]with a minimum OTU size of 20 sequences, then aligned and taxonomy assigned with the Human Oral Microbiome Database (HOMD) [23]as reference.

For alpha (within-sample) diversity, OTU tables were rarefied to 236000 for the *in vitro* culture samples and 74,000 reads for the saliva samples, and number of observed OTUs per sample as well as phylogenetic diversity per sample was calculated.

### Statistical analysis

Significance of difference between average value was analyzed by *t* test using MS Excel

## Results

### Antibacterial activity of licorice extract against *S*. *mutans in vitro*

**Fig 1:**
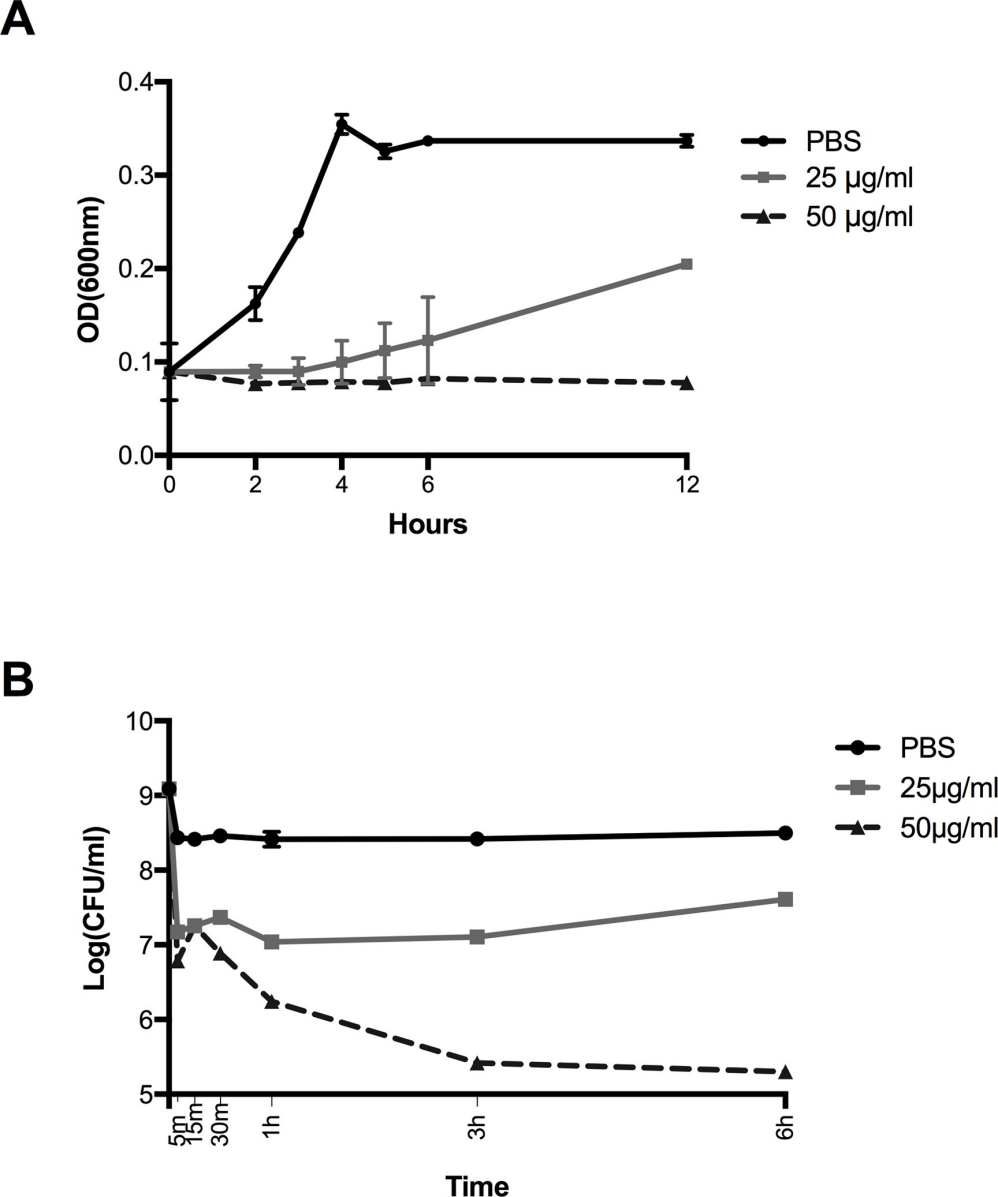
Antibacterial activity of licorice extract against *S*. *mutans in vitro*. A) Effect of licorice extract on growth of *S*. *mutans*. Licorice or equal volume of phosphate buffered saline (PBS, negative control) was added to the cultures at Time 0. Optical density was measured at 600nm at indicated time points. B) Killing activity of licorice extract against *S*. *mutans*. Cultures were exposed to licorice or phosphate buffered saline (PBS, negative control) for indicated amount of time. CFUs were counted by serial dilution and plating. For both A and B, values represent average of 2 biological replicates (with 2 technical replicates for each biological replicates). Error bars = SD. Where no error bars are visible, length of error bar is shorter than symbol.

We found the minimum inhibitory concentration (MIC) of licorice extract to be 25 μg/ml against *S*. *mutans*, which is consistent with pervious finding[16]. Next, we performed a growth curve assay, in which 25 μg/ml or 50 μg/ml (representing 1x and 2x the MIC, respectively) was added to the culture media, and *S*. *mutans* growth was monitored over time. 25 μg/ml of licorice extract dramatically hindered the growth of *S*. *mutans* compared to the saline control, while 50 μg/ml prevented growth almost completely (Fig 1A). The kill assay showed that exposure to 25 μg/ml of licorice extract led to a 98.8% decrease in viable bacteria after the first 5 min. 50 μg/ml killed 99.5% of bacteria after 5 min and ultimately resulted in 99.9% killing after 3 hr (Fig 1B).

### Effect of licorice extract on an *in vitro* multispecies oral microbial community

**Fig 2:**
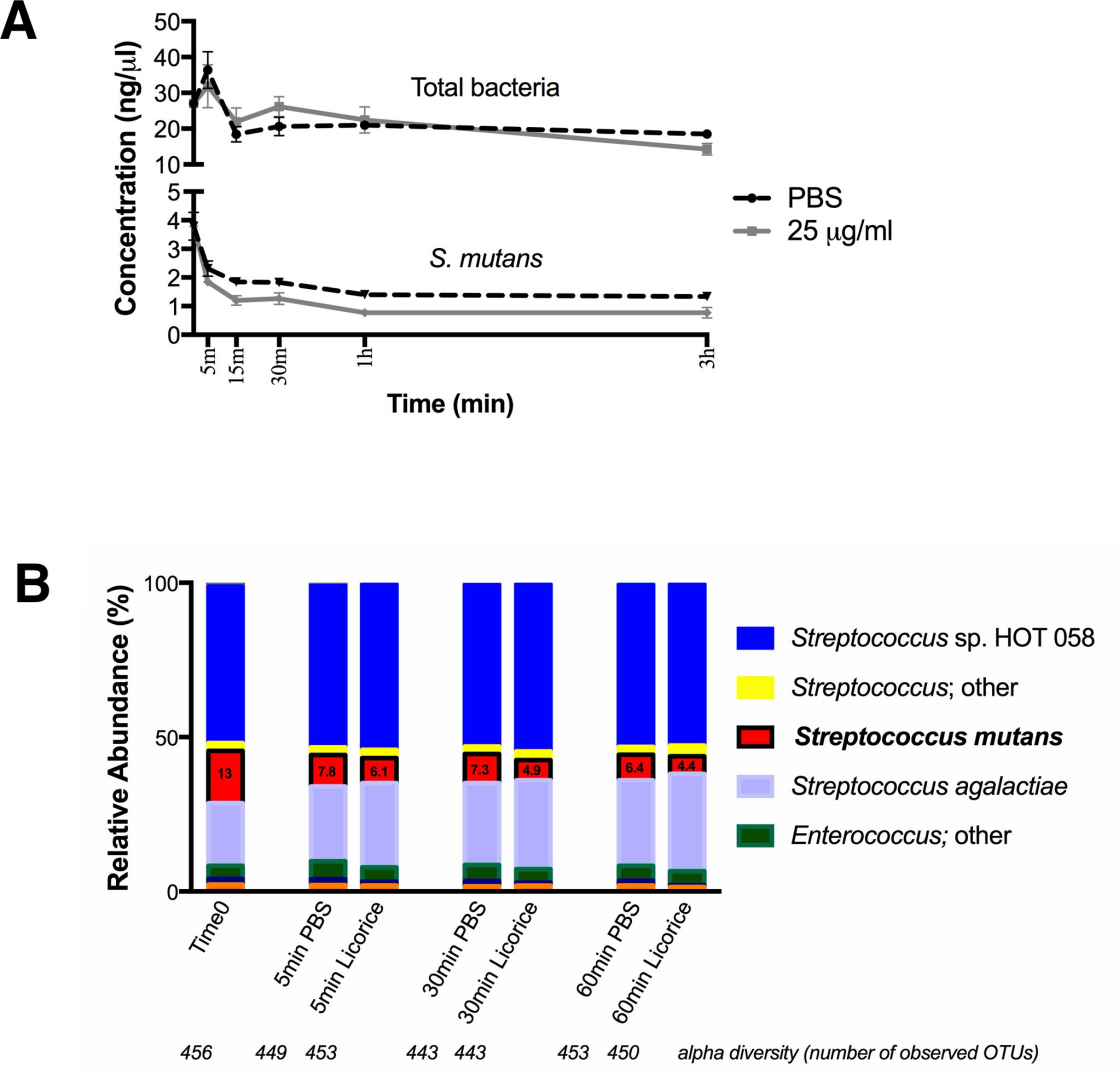
Effect of licorice extract against an *in vitro* multispecies oral microbial community. A) Concentration of total bacterial DNA and *S*. *mutans* DNA from each time point of a kill assay. Kill assay was performed by exposing the “*S*. *mutans-*enhanced*”* community culture to licorice or phosphate buffered saline (PBS, negative control) for indicated amount of time. To ensure that only viable bacteria were included in the analysis, at each time point cultures were pelleted, washed thoroughly, and regrown in fresh media (as described in Materials and Methods) before DNA extraction. Quantitative PCR was performed using *S*. *mutans-*specific primers and universal bacteria primers (“Total bacteria”) and used for determining *S*. *mutans* and total bacteria DNA concentration as described in Materials and Methods. Values represent average of 2 independent experiments. Error bars = SD. Where no error bars are visible, length of error bar is shorter than symbol. B) Results from 16S rRNA gene sequencing at samples from each time point. Stacked bar graphs represent the relative abundance of species detected in each sample. Bolded numbers on the graph indicate the percent relative abundance of *S*. *mutans*. Alpha diversity (number of observed OTUs) was calculated at a defined sequencing depth of 236,000 reads.

We utilized our well-developed *in vitro* oral microbiome model system [24] to test the effects of licorice extract on the oral microbial community. This microbial community was developed using pooled saliva from healthy adults and while it is a very good representation of the oral community, it contains a low abundance of *S*. *mutans* (0.02%). Since we were interested in the possible *S*. *mutans-*specific effect of the licorice extract, we added pure *S*. *mutans* culture to the community culture prior to testing, an approach that has been previously successfully used to study the efficacy of a targeted antimicrobial peptide in removing *S*. *mutans* [25]. The resulting “*S*. *mutans-* enhanced” community culture allowed us to more easily observe the effect of licorice treatment on *S*. *mutans* specifically and within the context of the entire oral community. We performed a kill assay on this culture, in which 25 μg/ml of the licorice extract was added to the media and then removed at different time points through centrifuging and washing. Fresh media without licorice was then added and cultures were incubated overnight before DNA was extracted. This period of regrowth ensured that only viable bacteria contributed to the total DNA used for analysis. We used qPCR to initially assess the amount of *S*. *mutans* DNA present in the community after licorice treatment compared to the total bacterial DNA present. Results show that, while the concentration of *S*. *mutans* decreased under exposure to the licorice extract compared to the negative control, the concentration of total bacteria is not affected (Fig 2A). This suggests a more specific killing effect of the licorice on *S*. *mutans* and not widespread bactericidal effects.

To further investigate the effects of licorice treatment on the diversity of the *in vitro* microbial community, we performed 16S rRNA gene sequencing on select time points of the kill assay described above. Diversity of the samples (as determined by calculating the number of observed OTUs at a defined sequencing depth) exposed to licorice did not change compared to the saline control, and licorice had little effect on the overall composition of the community. The initial relative abundance of *S*. *mutans* in the community was 13*%*, which decreased to 6.1% after 5 min exposure to licorice and to 4.4% after 60 min. This represents a 22% and 30% more decrease, respectively, compared to the saline control (Fig 2B).

Other *Streptococcus* species present in the community were not negatively affected by the licorice treatment; all other *Streptococcus* species combined did not decrease in abundance compared to the control (after 5 min: 79.1% relative abundance of all *Streptococcus* species combined present in control culture vs. 83.4% in licorice-treated; after 30 min: 81.2% in control culture vs. 85.6% in licorice-treated.)

### Lollipops containing licorice extract decreased *S*. *mutans* levels in a pilot clinical study in children with high caries risk

A total of 37 children were enrolled in the study, 20 boys and 17girls, with an average age of 4 years 10 months. 23 high-risk children (defined by antibody-detected levels of *S*. *mutans*>5x10^5^ cells/ml) received lollipops and oral health care counseling. 14 children received oral health care counseling only. Due to insufficient saliva collection, medication uptake during the study, or unable to comply with the treatment, 6 participants in the treatment group and 5 from the control group were excluded from the study. No side effects were observed. No caries increase was observed in either group. The dmft/dmfs scores are described in S1 Table.

**Fig 3:**
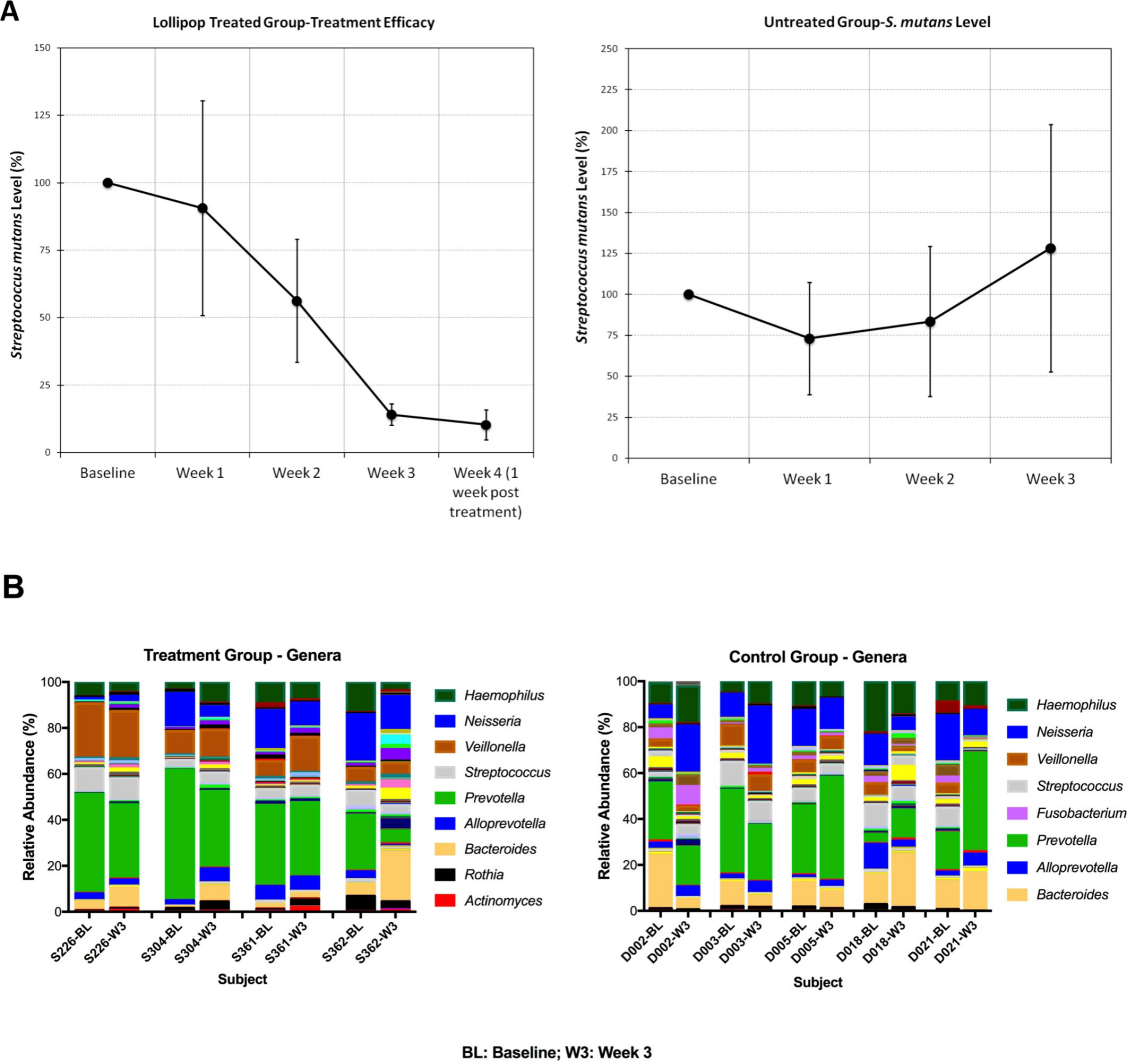
Lollipop treatment reduced salivary *S*. *mutans* level without disturbing overall microbiome. A) Salivary levels of *S*. *mutans*. Monoclonal antibody-based quantification of salivary *S*. *mutans* in treatment group and control group. (B). 16s rRNA gene sequencing analysis showing the relative abundance of genus level taxa detected in saliva samples from treatment group and control group.

Monoclonal antibody-based identification and quantification of *S*. *mutans*[20]was performed on saliva samples taken from each subject at baseline, and 1, 2, and 3 weeks after starting the study, and results were listed in detail in S2 Table. Changes of salivary *S*. *mutans* levels of each subject were shown in S1 Fig. A pooled level was calculated for each visit (Fig 3A). In children using the licorice-containing lollipops 2x/day, levels of *S*. *mutans* decreased dramatically (>80% reduction in bacterial count) over the course of the study, while no decrease was detected in the untreated group (Fig 3A).

### 16S rRNA gene microbiome analysis in select study subjects

To investigate the effects of the licorice extract-containing lollipops on the entire oral microbiome, we performed 16S rRNA gene sequencing on baseline and 3-week saliva samples from 5 subjects selected from each group. After sequencing, data from 1 subject from the treatment group had to be excluded due to insufficient reads. The mean number of sequences per sample was 122,938 after quality filtering. Analysis revealed a total of 8 phyla, 120 genera, and 287 species-level OTUs across all samples.

Phylogenetic diversity of each sample was calculated at a defined sequencing depth of 74,000 reads. Generally, diversity either increased or changed minimally in the treatment group. Alpha diversity [26] results showed overall there was slightly higher phylogenetic diversity at baseline in the control group (average 86) compared to the treatment group (average 66) (S3 Table). This could be due to the fact that even though subjects from the control and treatment group carried similar level of salivary *S*. *mutans* (S2 Table) at baseline, the control group had low dmft/dmfs (average 1.2/2.2) compared to that of the treatment group (average 26.8/29), indicating the control group is clinically healthier than treatment group despite similarly high “caries risk” level based on salivary *S*. *mutans* counts. By the end of third week, there was an average 7.3 (12.5%) increase in phylogenic diversity in the treatment compared to baseline, which brought the overall diversity up (average 74) to the level of control group (average 80) (S3 Table). Particularly, two subjects (S226 and S304) from the treatment group had the lowest baseline diversity of any subject in the study, and also experienced the highest increases in diversity at the 3-week timepoint (S3 Table). Intriguingly, no taxa were significantly differentially abundant in the control group compared to the treatment group after controlling for multiple comparisons. Meanwhile, for the treatment group, no significant change in microbial diversity was monitored between baseline samples and those taken at week 3 (Fig 3B). Even though we were not able to obtain the relative abundance data on *S*. *mutans* due to the inability of 16S rRNA gene V3-V4 region in differentiating *S*. *mutans* with other streptococcus species, the reduction in *S*. *mutans* count using antibody-based method in the treatment group following lollipop usage suggested that other non-*S*. *mutans* streptococcus spp. are not affected and even increase in abundance.

The data also suggest that licorice extract in the lollipops does not have broad-spectrum killing activity and can preserve the diversity of the oral microbiome or even modulate the microbiome resulting in a community closer to representing healthy controls.

## Discussion

In China, the prevalence of ECC is extremely high, while the care index is very low. For caries prevention, one approach is to prevent *S*. *mutans* from accumulating to pathologic levels through topical application of antimicrobial agents[27].However, the effective delivery of therapeutic agents is difficult and challenging, as most are cleared from the mouth so rapidly that they cannot exert their maximum therapeutic potential[28]. The lollipop delivery form presented here is simple and effective, and helps to prolong the exposure time of the agent to the tooth surfaces. In a previous study, lollipop delivery of therapeutic agents helped to reduce the intake of sugar and was widely accepted by children, as it does not require behavior changes for children fond of sweets [16]. The lollipop ingredients are all FDA-approved for human consumption, and unlike fluoride products or antibiotic agents, there is less risk of adverse effects due to overuse.

Our *in vitro* data using *S*. *mutans* mono-species are in agreement with previous study[16, 29], showing effective killing of Glycyrrhizol A-containing licorice root extract against *S*. *mutans* (Fig 1). We also tested a well-established *in vitro* multispecies oral microbial community spiked with *S*. *mutans* to evaluate the efficacy of licorice extract in targeting *S*. *mutans* within a microbial community. Although SHI medium was able to support a multispecies community with high diversity, it does not support the growth of *S*. *mutans* very well, which may explain the decrease of *S*. *mutans* even in the non-treatment group. In order to support the growth of *S*. *mutans* and allow it to be maintained in the community over the course of the short (6 h) experiment, 15 mM of sucrose was added to the SHI media. Sucrose addition does come with a trade-off, as it lowers diversity dramatically. However, although not a perfect model, the resultant “*S*. *mutans*-enhanced’ culture represents a diseased microbial community with increased *S*. *mutans* load and reduced microbial diversity[30], which has been successfully employed to evaluate the efficacy of C16G2, a specifically-targeted antimicrobial peptide (STAMP) that selectively kills *S*. *mutans*[25]. Results of the kill-assay showed licorice extract treatment induced more significant reduction in relative abundance of *S*. *mutans* but not the overall *Streptococci* population (Fig 2). This is consistent with our previous finding that when using STAMP C16G2 for targeted removal of *S*. *mutans*, the decrease in abundance of *S*. *mutans* was coupled with increase in other *Streptococcus spp*.[25]. Furthermore, our *in vitro* data demonstrated that licorice extracts did not significantly impact overall microbial diversity.

We further investigated *in vivo* the efficacy of herbal lollipop containing the same licorice extract as tested *in vitro*. In the treatment group, the pooled salivary *S*. *mutans* level decreased significantly during the 3-week uptake of licorice-containing lollipops, with the reduced level stable in the follow-up visit (week 4) after the conclusion of the treatment (Fig 3A), which suggests long-term efficacy in *S*. *mutans* inhibition. This is consistent with our previous study showing that, targeted antimicrobial therapy using STAMP C16G2 against *S*. *mutans* within an *in vitro* multispecies microbial community established protective non-cariogenic biofilm and reduced subsequent *S*. *mutans* infection [31]. These data suggest that the shift in microbiome as a result of treatment may help maintain a healthy or “normal” biofilm and have long-term effects on preventing recovery or re-colonization of *S*. *mutans*. The average salivary level of *S*. *mutans* in the control group decreased sharply after the first visit, but gradually relapsed in the following 2 weeks. This could be explained by the oral health education they received during the first visit, in which they received tooth-brushing guidance and dietary recommendations. However, this was not sustainable.

New paradigm in human-associated microbiome suggests that majority of the commensal species are indispensable for maintaining a balanced microbial community and contribute positively toward host health. These new insights demand a new strategy for caries prevention. Instead of non-discriminative killing of oral microbiome which often leads to further deterioration of the condition, more targeted killing of cariogenic bacterium without affecting the overall microbial biodiversity would be preferred. Our data showed that, while usage of the lollipop significantly reduced the salivary *S*. *mutans* counts, the treatment not only maintained the microbial diversity, but in two individuals with very low alpha diversity at baseline, an increase in biodiversity was observed after treatment (S3 Table). These data suggested that licorice extract in the lollipops does not have broad-spectrum killing activity and can preserve or even increase the diversity of the oral microbiome which is often associated with increased health status. Our finding is consistent with the reports that licorice extracts exert antimicrobial efficacy towards oral *S*. *mutans*[16, 29]. Although the underlying mechanisms for more selective killing of *S*. *mutans* remains an intriguing question that warrants further investigation, our study provides corroborating evidence that a licorice extract-containing lollipop could represent a promising preventative measurement in fighting dental caries in high-risk children.

One limitation of the current study we would like to point out is that, due to parents’ unwillingness to use on their kids the products of no potential benefit, no placebo was used in the control group. However, based on the *in vitro* data showing the effectiveness of licorice extract in targeting *S*. *mutans* (Figs 1 and 2), as well as the similar trend observed between current (Fig 3) and previous *in vivo* study where a placebo group was included[17], it is most likely that the observed reduction in *S*. *mutans* counts after herbal lollipop treatment in this study is due to added licorice extract. Meanwhile, we controlled for the improved oral hygiene due to being enrolled in the study and receiving dental visits by having the control group undergo all 4 visits.

Our study showed that the short-term use of herbal lollipops may reduce cariogenic *S*. *mutans*, one of the primary risk factors in dental caries, while preserve oral microbial diversity. More long-term observation is needed to confirm efficacy in reducing the occurrence dental caries clinically.

## Conclusion

The twice-a-day use of herbal lollipops for 3 weeks significantly reduced the salivary *S*. *mutans* levels in Chinese preschool children. Domestic use of this lollipop should be considered as a simple and effective way to reduce the risk of dental caries in children.

## Supporting information

S1 Fig Changes of salivary *Streptococcus mutans* levels of each subject. (A) The treatment group;(B) The control group. S1 Fig was generated using data in S2 Table.
(TIF)

S1 Table dmft/dmfs record of included subjects. (DOCX)

S2 Table MAb-based assessments of salivary *S*. *mutans* concentration of each subject. (DOCX)

S3 Table Alpha diversity of saliva microbiome. (DOCX)
