# Supplementary figures and images for: Lollipop containing Glycyrrhiza uralensis extract reduces Streptococcus mutans colonization and maintains oral microbial diversity in Chinese preschool children
Source: PLoS One. 2019 Aug 23;14(8):e0221756. doi: 10.1371/journal.pone.0221756 (PMC6707631; doi:10.1371/journal.pone.0221756)

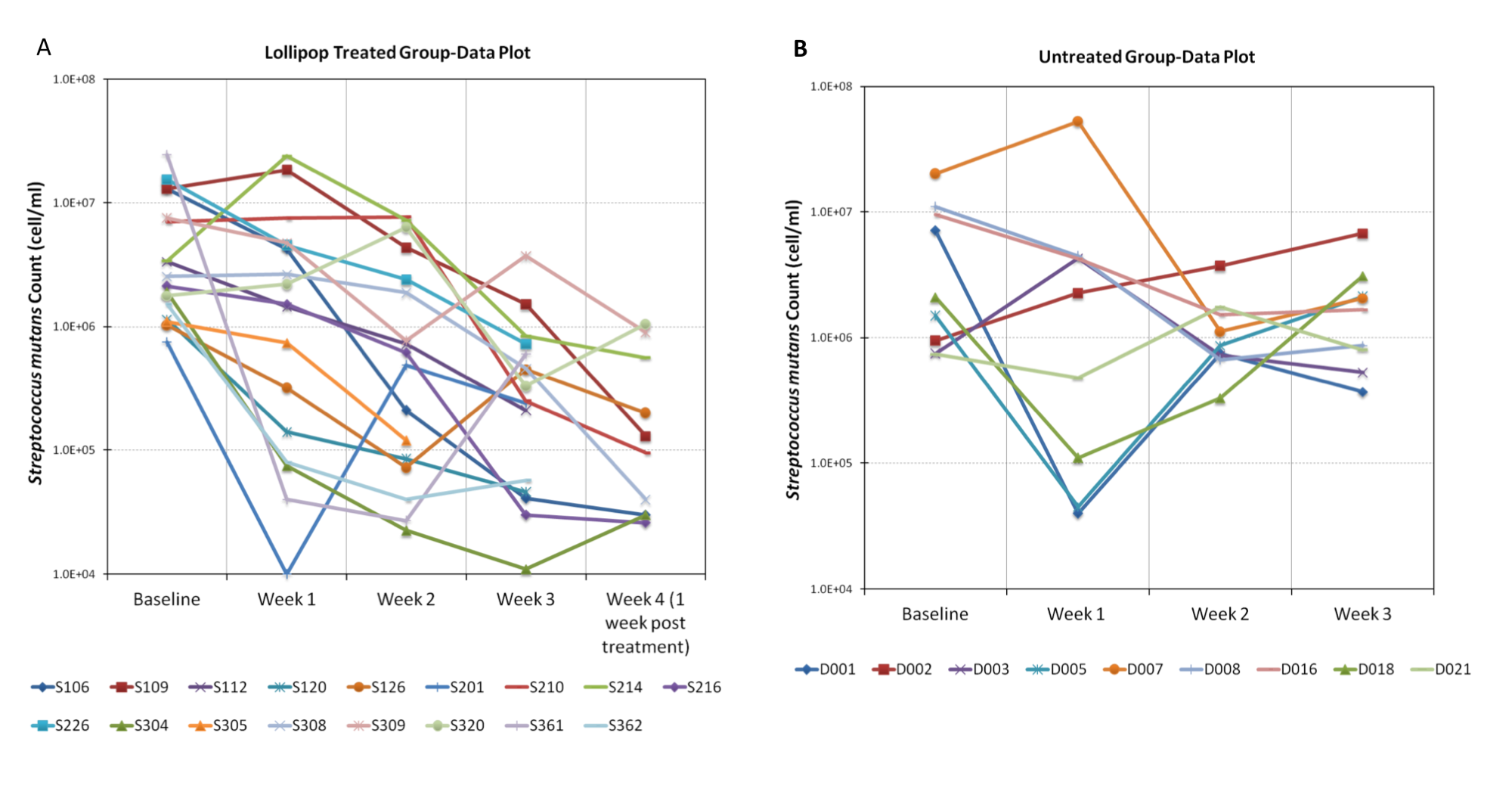

Supplement: S1 Fig — (A) The treatment group;(B) The control group. S1 Fig was generated using data in S2 Table. (TIF) [file pone.0221756.s001.tif]

**S3 Table: Alpha diversity of saliva microbiome**


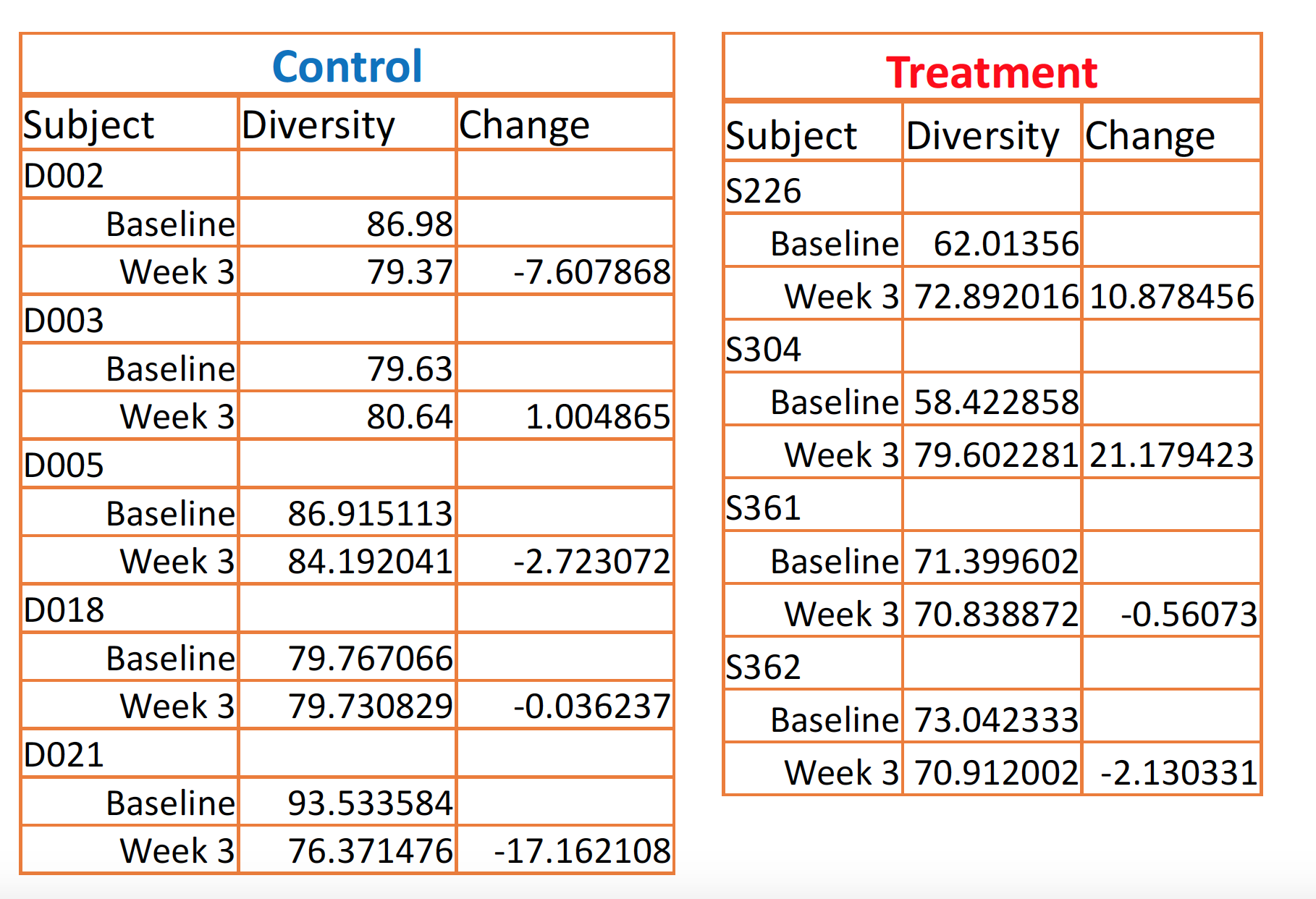

Supplement: S3 Table — (DOCX) [file pone.0221756.s004.docx]
