# Supplementary material for: Lollipop containing Glycyrrhiza uralensis extract reduces Streptococcus mutans colonization and maintains oral microbial diversity in Chinese preschool children
Source: PLoS One. 2019 Aug 23;14(8):e0221756. doi: 10.1371/journal.pone.0221756 (PMC6707631; doi:10.1371/journal.pone.0221756)
Supplement: S1 Table — (DOCX) [file pone.0221756.s002.docx]

**S1 Table. dmft/dmfs record of included subjects**

| Treatment Group | | | Control Group | | |
| --- | --- | --- | --- | --- | --- |
| Subject No. | dmft | dmfs | Subject No. | dmft | dmfs |
| S106 | 7 | 31 | D001 | 2 | 2 |
| S109 | 4 | 20 | D002 | 0 | 0 |
| S112 | 11 | 52 | D003 | 1 | 1 |
| S120 | 14 | 58 | D005 | 0 | 0 |
| S126 | 10 | 48 | D007 | 9 | 15 |
| S201 | 19 | 49 | D008 | 6 | 11 |
| S210 | 17 | 43 | D016 | 8 | 10 |
| S214 | 13 | 25 | D018 | 0 | 0 |
| S216 | 17 | 25 | D021 | 5 | 10 |
| S226 | 13 | 26 |  |  |  |
| S304 | 8 | 20 |  |  |  |
| S305 | 2 | 10 |  |  |  |
| S308 | 5 | 7 |  |  |  |
| S309 | 5 | 7 |  |  |  |
| S320 | 9 | 9 |  |  |  |
| S361 | 20 | 65 |  |  |  |
| S362 | 3 | 5 |  |  |  |
