# Supplementary material for: Lollipop containing Glycyrrhiza uralensis extract reduces Streptococcus mutans colonization and maintains oral microbial diversity in Chinese preschool children
Source: PLoS One. 2019 Aug 23;14(8):e0221756. doi: 10.1371/journal.pone.0221756 (PMC6707631; doi:10.1371/journal.pone.0221756)
Supplement: S2 Table — (DOCX) [file pone.0221756.s003.docx]

**S2 Table. MAb-based assessments of salivary *S. mutans* concentration of each subject**

| Group | Subject No. | Baseline | 1 week | 2 weeks | 3 weeks | 1 week Follow-up |
| --- | --- | --- | --- | --- | --- | --- |
| Treatment group | S106 | 1.3x10^7^ | 4.25x10^6^ | 2.1x10^5^ | 4.1x10^4^ | 3x10^4^ |
|  | S109 | 1.3x10^7^ | 1.84x10^7^ | 4.34x10^6^ | 1.52x10^6^ | 1.3x10^5^ |
|  | S112 | 3.35x10^6^ | 1.45x10^6^ | 7.26x10^5^ | 2.1x10^5^ | NA |
|  | S120 | 1.15x10^6^ | 1.4x10^5^ | 8.5x10^4^ | 4.6x10^4^ | NA |
|  | S126 | 1.03x10^6^ | 3.2x10^5^ | 7.24x10^4^ | 4.5x10^5^ | 2X10^5^ |
|  | S201 | 7.53x10^5^ | ＜1x10^4^ | 4.9x10^5^ | 2.4x10^5^ | NA |
|  | S210 | 7x10^6^ | 7.5x10^6^ | 7.7x10^6^ | 2.5x10^5^ | 9.5X10^4^ |
|  | S214 | 3.4x10^6^ | 2.39x10^7^ | 7.2x10^6^ | 8.4x10^5^ | 5.6X10^5^ |
|  | S216 | 2.13x10^6^ | 1.52x10^6^ | 6.2x10^5^ | 3x10^4^ | 2.6X10^4^ |
|  | S226 | 1.54x10^7^ | 4.55x10^6^ | 2.4x10^6^ | 7.25x10^5^ | NA |
|  | S304 | 1.9x10^6^ | 7.5x10^4^ | 2.25x10^4^ | 1.1x10^4^ | 3X10^4^ |
|  | S305 | 1.1x10^6^ | 7.42x10^5^ | 1.2x10^5^ | NA | NA |
|  | S308 | 2.55x10^6^ | 2.65x10^6^ | 1.88x10^6^ | 4.6x10^5^ | 4X10^4^ |
|  | S309 | 7.55x10^6^ | 4.75x10^6^ | 7.75x10^5^ | 3.72x10^6^ | 9X10^5^ |
|  | S320 | 1.78x10^6^ | 2.2x10^6^ | 6.31x10^6^ | 3.3x10^5^ | 1.05X10^6^ |
|  | S361 | 2.45x10^7^ | 4x10^4^ | 2.7x10^4^ | 6x10^5^ | NA |
|  | S362 | 1.52x10^6^ | 8x10^4^ | 4x10^4^ | 5.7x10^4^ | NA |
|  | | | | | | |
| Control group | D001 | 7.1x10^6^ | 4X10^4^ | 7.5X10^5^ | 3.7X10^5^ | NA |
|  | D002 | 9.5x10^5^ | 2.26X10^6^ | 3.72X10^6^ | 6.75X10^6^ | NA |
|  | D003 | 7.5x10^5^ | 4.27X10^6^ | 7.25X10^5^ | 5.3X10^5^ | NA |
|  | D005 | 1.5x10^6^ | 4.5X10^4^ | 8.7X10^5^ | 2.13X10^6^ | NA |
|  | D007 | 2.02x10^7^ | 5.25X10^7^ | 1.12X10^6^ | 2.06X10^6^ | NA |
|  | D008 | 1.1x10^7^ | 4.47X10^6^ | 6.7X10^5^ | 8.7X10^5^ | NA |
|  | D016 | 9.5x10^6^ | 4.23X10^6^ | 1.53X10^6^ | 1.67X10^6^ | NA |
|  | D018 | 2.1x10^6^ | 1.1X10^5^ | 3.3X10^5^ | 3.1X10^6^ | NA |
|  | D021 | 7.4x10^5^ | 4.8X10^5^ | 1.75X10^6^ | 8X10^5^ | NA |

**NA: not available**
